# In and out among the Hospitals

**Published:** 1888-10-06

**Authors:** 


### In and Out Among the Hospitals.

By a Secretary of Ten Years' Standing.

Copies of Annual Reports and Rules, Accounts of Meetings, Changes in the Staff, &c., should be addressed The Editor, The Lodge, Porchester Square, W.

### THE ISLE OF MAN GENERAL HOSPITAL AND DISPENSARY.

This is a thriving institution, doing a large amount of good with the beds at its disposal. Through the munificence of Mr. and Mrs. Noble, the latter of whom has just died, additional bed accommodation has been added, and there are now 24 available. During the twelve months ending June last, the daily average number has been 18, and the total number of in-patients 181. This number, which is a considerably larger than in former years, is attributable to the increase in population, and as there is no other general hospital, patients come from all parts of the island. The medical officer, however, points out in his report that many patients apply for admission who are not what are generally considered fit cases for hospital treatment, as many of them could, in his opinion, pay for medical treatment outside. On turning to the rules we find that they are framed so as almost to encourage this state of affairs, for the hospital is not free, but patients have to obtain letters of recommendation, and subscribers can recommend patients to remain from three weeks to two months, according to the amount of their subscription. A patient who makes a collection of out-patients' letters can obtain admission as an in-patient for a longer or shorter period according to the number of out-patients' letters over ten that he may have obtained. Almost everybody on the island subscribes to the hospital; most of the subscriptions are very small, but they are collected by private individuals, who undertake to collect in a district or single street, and so a house-to-house collection is made every year. The committee record with regret the loss the hospital sustains in the resignation of the house surgeon, Dr. Roxburgh, who, after a long service, has obtained an appointment at Glasgow. Two thousand and ten out-patients were also treated. Deducting 1*s.* for each of these, and extraordinary expenditure, it is found that the cost per bed occupied was £52 13*s.* 10*d.* The total income was £1,422 0*s.* 11*d.*, and the expenditure £1,415 15*s.*

### SUSSEX COUNTY HOSPITAL.

This is one of the most fortunate of county hospitals. Being situated in Brighton, it enjoys a prominence that brings it to the notice of many Londoners and other wealthy visitors, and very deserving it is of their sympathy and support. In every respect it is kept up to the times, and only last year various extensive alterations and improvements were made in the building. The new nurses' home is not the least valuable of its latest acquirements. This has been placed under the charge and management of Miss Scott, the matron, and it is anticipated, and not without reason, that the home will be a source of revenue, not only directly but indirectly, to the institution. Invalids who visit Brighton will many of them avail themselves of the valuable services of the hospital-trained nurse, and in no way do people hear more of hospitals or of the good they do than from these devoted and trusted helpers who come to our aid when we are ill. From them we hear of hospitals in a light that is often unfamiliar to us, for a nurse whose heart is in her work thinks there is no place like the hospital at which she received her training, and it is as if talking of a friend when she tells her patient all about it and the work that is done therein. The last published report states that there were 1,174 in-patients and 4,754 out-patients treated during the year, at an expenditure of £12,382. Deducting the expenditure on buildings and the cost of out-patients, it is found that £71 14*s.* 7*d.* was spent on each bed occupied, the daily average number being 131. Mr. R. Black has been appointed assistant surgeon, vice Mr. Uhthoff, resigned, and Dr. Richardson assistant physician, vice Dr. Shadwell.

### THE GERMAN HOSPITAL.

There are few hospitals which have as many Royal supporters, or which are so well managed as this institution. The charity is patronised by the Grand Dukes of many German States and other complex dignitaries of the Teutonic race. His Royal Highness the Duke of Cambridge has taken special interest in this hospital, and has presided at the festival dinner no less than eighteen times; last year, when he was in the chair, the donations amounted to £2,962. The hospital, though primarily for the benefit of German-speaking people, deserves well of us, for out of 1,470 patients treated in the wards 520 were English. Most of these were cases of accident, and as such are some of the most expensive patients in the hospital. There is a convalescent home containing 17 beds in connexion with the charity, and two beds always available at the seaside. Dispensaries are also maintained in the City Road and at 28, Guilford Street, W.C., at both of which places patients are seen twice a week. The committee speak in very high terms of the nursing sisters who come from the Deaconesses' Institution at Darmstadt. There were 17,122 patients treated in the out-patients' department and at the dispensaries in 1887, while the daily average number in the wards was 110. Deducting one shilling for each out-patient and the extraordinary expenditure, it is found that the cost per bed occupied was £67 8*s.* 11*d.* The total receipts were £9,144 11*s.* 3*d.*, and the expenditure £8,271 19*s.* 4*d.*

### COUNTY DONEGAL INFIRMARY.

An interesting report is published by the governors of this charity. From it we find that an average of 24 beds were occupied daily during the last twelve months. The cases treated have been serious, and include a large number of operations. A county presentment of £1,000 is paid annually to the infirmary at the Assizes, and this forms the principal income of the charity. The total receipts were £1,142 12*s.* 1*d.*, and the expenditure £859 9*s.* 3*d.*; deducting extraordinary expenditure (there not appearing to be any out-patients) the cost per bed occupied was £35 16*s.* 2*d.* An excellent diet table is also published, showing that a liberal supply of food is given to the inmates, while a special diet is provided for Fast-days if the patients desire it.

